# Correction: Mild decarboxylation of neat muconic acid to levulinic acid: a combined experimental and computational mechanistic study

**DOI:** 10.1039/d5ra90069g

**Published:** 2025-06-09

**Authors:** Siddhant Bhardwaj, Deep M. Patel, Michael J. Forrester, Luke T. Roling, Eric W. Cochran

**Affiliations:** a Department of Chemical and Biological Engineering, Iowa State University Ames IA 50011 USA ecochran@iastate.edu +1-515-294-0625; b Center for Biorenewable Chemicals (CBiRC) Ames IA 50011 USA

## Abstract

Correction for ‘Mild decarboxylation of neat muconic acid to levulinic acid: a combined experimental and computational mechanistic study’ by Siddhant Bhardwaj *et al.*, *RSC Adv.*, 2024, **14**, 39408–39417, https://doi.org/10.1039/D4RA05226A.

The authors regret the omission of a reference from the original manuscript, which should have been included in addition to ref. 35–41 in the sentence below. This reference is shown below as ref. 1.

“MA serves as a key platform chemical that readily affords a plethora of critical commodity chemicals, including adipic acid, terephthalic acid, ε-caprolactam, and 1,6-hexamethylene diamine, and novel monomers like cyclohex-1-ene-dicarboxylic acid (CH1DA).^35–41^”

The Royal Society of Chemistry apologises for these errors and any consequent inconvenience to authors and readers.
